# A study protocol for a randomised controlled trial evaluating the use of information communication technology (WhatsApp/WeChat) to deliver brief motivational interviewing (i-BMI) in promoting smoking cessation among smokers with chronic diseases

**DOI:** 10.1186/s12889-019-7417-6

**Published:** 2019-08-09

**Authors:** William Ho Cheung Li, Ka Yan Ho, Katherine Ka Wai Lam, Man Ping Wang, Derek Yee Tak Cheung, Laurie Long Kwan Ho, Wei Xia, Tai Hing Lam

**Affiliations:** 1 School of Nursing, University of Hong Kong, 21 Sassoon Rd, Pokfulam, Hong Kong SAR; 2 School of Nursing, The Hong Kong Polytechnic Unversity, Hung Hom, Hong Kong SAR; 3 School of Public Health, University of Hong Kong,, Pokfulam, Hong Kong SAR

**Keywords:** Smoking cessation, Chronic disease, General health, Motivational interviewing, Information communication technology, Randomized controlled trial

## Abstract

**Background:**

The recent development of smoking cessation interventions for smokers with chronic diseases has focused heavily on brief interventions. However, these interventions are too brief to make an impact on these smokers, especially when most of them are without any intention to quit. Previous studies showed that smokers who did not want to quit might be interested in changing other health behaviours. Also, once people engage in a health behaviour, they are found more likely to change other unhealthy habits. Hence, a general health promotion approach could be a feasible approach to motivate smokers who do not want to quit to first engage in any desirable health behaviour, and later quit smoking when they intend to do so. This study aims to determine the potential efficacy and effect size of such intervention approach in promoting smoking cessation for smokers with chronic diseases.

**Methods:**

This is a randomized controlled trial. A convenience sample of 60 smokers with chronic diseases will be randomly assigned into either experimental (*n* = 30) or control group (*n* = 30). Smokers in the experimental group will receive an individual face-to-face brief motivational interviewing (MI) with generic advice on selected health behaviour. More brief MI messages will be delivered to them via WhatsApp/WeChat for 6 months. For subject in the control group, they will be asked to indicate their desirable health-related practice. However, no MI and booster interventions will be given. All subjects will complete a questionnaire at 1, 3, 6 and 12 months. Subjects abstinent from cigarettes at 12 months will perform a biochemical validation. The primary outcome is biochemically validated smoking abstinence at 12 months. Effect size of the intervention will be estimated by the odd ratios using intention-to-treat.

**Discussion:**

This is the first study to determine the potential efficacy for the use of a personalized general health promotion approach in promoting smoking cessation for smokers with chronic diseases. If our proposed intervention is effective, we will able to assist smokers with chronic disease to quit smoking and change their health behaviour simultaneously.

**Trial registration:**

CinicalTrials.gov NCT03983330 (Prospectively registered), registered on June 12, 2019.

## Background

Smoking has harmful effects on nearly every organ of the body and causes 7 million deaths worldwide every year [1, 2]. Although the prevalence of daily cigarette smoking in Hong Kong has decreased from 23.3% in 1982 to 10.5% in 2015, there are still 641,300 daily smokers [3] and 400,000 hospitalisations per year that are attributable to smoking [4]. Having a disease and requiring medical attention present an excellent ‘teachable moment’ and opportunity for initiating smoking cessation in patients, because they will be more likely to be motivated to alter their habits and improve their health. However, cigarette smoking is addictive and quitting is very difficult, with a high rate of relapse, particularly among patients with chronic diseases [5].

During the past decade, several randomised controlled trials have been conducted to promote smoking cessation for smokers with chronic diseases, including cardiac [6], type 2 diabetes mellitus [5], and cancer [7]. It was observed that many smokers with chronic diseases had a long smoking history, high nicotine dependency, no quit attempt, and no intention to quit. Results of these studies indicated that about 68% smokers with cardiac diseases, 70% with diabetes mellitus and 73% with cancer recruited in Special Out-Patient Clinics (SOPC) were still in the pre-contemplation stage. These studies also revealed that most smokers with chronic diseases perceived more barriers in quitting than benefits of quitting [7]. Nevertheless, the recent development of smoking cessation interventions for smokers with chronic diseases has focused heavily on brief interventions, including stage-matched smoking cessation advices [5, 7, 8]. However, these interventions could be too brief and inadequate to make a great impact on chronic smokers [8]. In addition, the use of strong warnings to communicate the risk of continued smoking might not be accepted by some chronic smokers [7]. Hence, it is imperative for healthcare professionals to develop and evaluate a more innovative intervention to enhance the effectiveness in promoting smoking cessation for smokers with chronic diseases. Most importantly, the new strategy should have good potential implementation in many clinical settings.

On the other hand, smoking has been found to be associated with physical inactivity [9], unhealthy diet [10], and drinking [11]. The interrelationship of health behaviours suggests that there could be a higher level of attribute that determines such behaviours together. Our previous studies [12, 13] showed that people with a general intention to promote their health are more likely to engage in desirable health-related lifestyle practices. In addition, research results showed that people once engaged in any desirable health-related lifestyle practices would progressively move to later stages of change for other health behaviours [12, 13]. Based on this concept, a general health promotion approach could be a feasible and effective approach to motivate smokers with intention to promote health to first engage in any desirable health-related lifestyle practices that are chosen by individual smokers, such as regular physical activity and healthy diet. It is anticipated that once they are engaged in any desirable health-related lifestyle practice they will eventually be more motivated to quit smoking.

### Conceptual framework

Our proposed intervention will be developed according to and guided by the (i) foot-in-the-door technique and (ii) brief motivational interviewing (MI).

#### Foot-in-the-door technique

The technique was introduced by Freeman and Fraser [14] for investigating individuals’ compliance without pressure. It emphasizes the notion that individuals who are induced to comply with a smaller and easier request initially are more likely to comply with and achieve a larger request [14]. Their agreements for the first requests or targets increase their confidence and alter their attitudes towards themselves that they are capable and willing to take further actions.

#### Brief MI

MI was originally developed in the field of addictions and found to be transferable to other health-related behaviours including smoking cessation [15, 16]. However, traditional MI generally takes over 30 min to implement and is not feasible in busy clinical settings. A brief MI [17] was thus developed with the aim to provide brief consultations in medical settings. Brief MI shares the same core as MI that individuals are advocates to initiate and continue behavioural change, and yet often in a state of ambivalence with fluctuating motivations before the behavioural change. Brief MI and MI therefore focus on using specific techniques to explore and resolve the ambivalence, develop discrepancies between individuals’ core belief and the behaviour of not engaging in desirable health-related lifestyle practice, consequently enhancing the confidence and motivation in the behavioural change. Brief MI emphasizes on adopting shorter and simpler strategies, which include opening strategy, a typical day, the good things and the less good things, providing information, the future and the present, exploring concerns and helping with decision-making.

#### Incorporating foot-in-the-door technique and brief MI to initiate a personalized general health promotion using information communication technology

The foot-in-the-door technique will be incorporated into the conceptual basis of the intervention to facilitate the recruitment process and enhance compliance of smokers. According to previous studies, most smokers with chronic diseases have had no intention to quit [5–7]. However, they were found to be interested in promoting their health [12, 13]. Through asking these smokers to identify, change and engage in their desirable health-related behaviours (a smaller and easier request), it is expected that they will be more motivated to quit smoking (a larger request) in later time.

To guide the process of behavioural change, brief MI will be used to provide a strong theoretical framework to the intervention. Most importantly, we use Information Communication Technology, i.e. WhatsApp/WeChat, to provide brief MI via smart phone. Accordingly, we will start the conversation in WhatsApp/WeChat messengers by asking the smokers about their current stress and lifestyles that are related to health, so as to establish a collaborative relationship and have a general understanding about the smokers (opening strategy). To further comprehend the context of not engaging in the desirable health-related lifestyle practice, we will review a typical day of the smokers (a typical day) and ask about the pros and cons of not adopting the desirable health-related lifestyle practice (the good things and the less good things). The smokers will receive further information about that if he/she is interested (providing information). To elicit the discrepancy, the smokers will be encouraged to talk about his/her ideal future, compare the past and the present (the future and the present). We will also listen, explore and summarize the concerns with the smokers (exploring concerns). To resolve the ambivalence, we will further describe what others have done in a similar situation, and present options while not pushing the smokers for decision making (helping with decision-making). Importantly, the confidence will be enhanced throughout the process of giving health advice by encouraging the smokers to be aware of any resource that enables and facilitates the change. By using such a strategic approach, the smokers will realize the discrepancy, be motivated and discuss an action plan for adopting the new desirable health-related lifestyle practice.

### Aims

A study will be conducted to determine the potential efficacy and effect size of a personalized general health promotion approach using Information Communication Technology (WhatsApp or WeChat) to deliver a brief MI in promoting smoking cessation among smokers having follow-up in a SOPC.

## Methods

### Study design

**Fig. 1:**
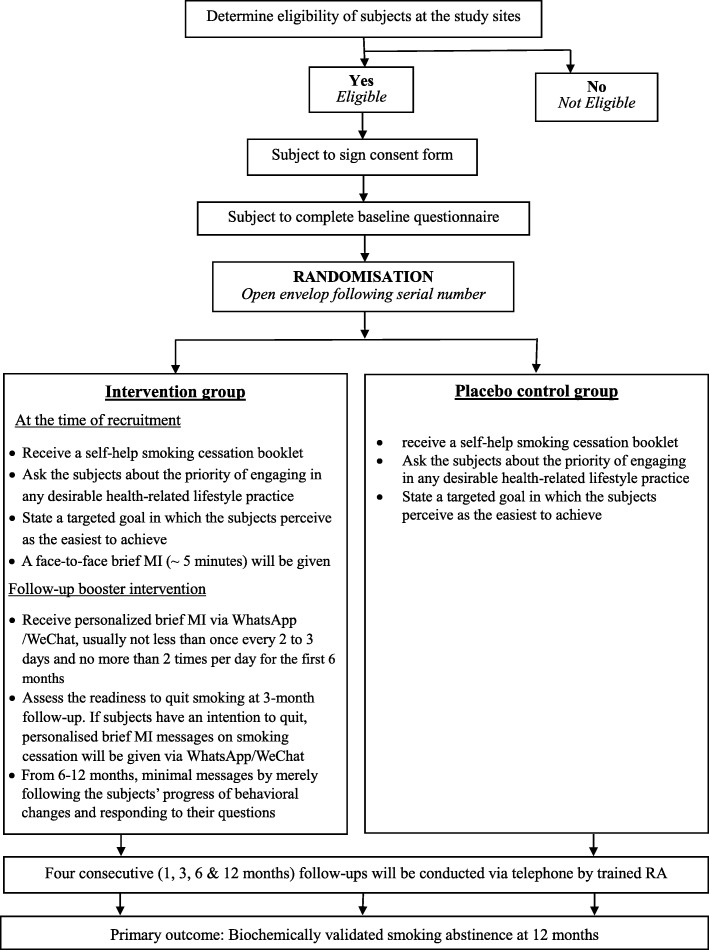
Study protocol (CONSORT statements)

This protocol (date: 20 December 2018) is original. The study will be completed within 18 months (from 1st June 2019 to 30th November 2020). A randomised controlled trial, with two-group pre-test and repeated post-test, between subjects design will be adopted following CONSORT statements (Fig. 1).

### Subjects

Hong Kong Chinese smokers with chronic diseases who have medical follow-up in a SOPC and fulfil the following inclusion criteria will be invited to participate: (1) aged 18 years or above, (2) able to speak Cantonese and read Chinese, (3) no intention to quit smoking (pre-contemplation stage), but are willing to take action to promote health, (4) have a smart phone and able to use instant messaging tool (e.g. WhatsApp, WeChat) for communication, and (5) willing to receive health promotion advices via WhatsApp/WeChat in the smart phone throughout the study. The exclusion criteria are: (1) unable to give informed consent or participate in our intervention due to impaired mental status, cognitive impairment, or communication barrier, and (2) participation in other smoking cessation programmes or services.

### Randomisation and blinding

The method of simple complete randomisation will be adopted. The subjects will be randomly allocated into one of the two groups: the control group or the intervention group. The randomisation will be performed by a research assistant who will open a serially numbered, opaque and sealed envelope (SNOSE) with a card inside indicating the randomly allocated group. The random numbers used for group assignment will be generated using a personal computer by another research assistant who does not involve in subject recruitment. Since the intervention is not a usual practice in the SOPC, we cannot completely blind the subjects. However, a single-blind approach will be adopted, with all outcome assessors and data analysts will be blinded to the group assignment.

### Sample size calculation

To determine the efficacy of using a personalized general health promotion approach in promoting smoking cessation for smokers with chronic diseases, we will recruit 60 smokers (30 in the intervention and 30 in the control groups) having medical follow-up in a SOPC in this study.

Power analysis will not be adopted for calculating the sample size as we have found no similar intervention in the literature. However, with the available resources and the proposed timeframe, we will be able to recruit 60 subjects. We therefore propose to have the sample size of 60 for this study, with 30 in the experimental group and 30 in the control group.

### Intervention group

#### In SOPCs

After completing the baseline questionnaires, the trained research assistant will first ask the subjects about the priority of engaging in any desirable health-related lifestyle practice as identified in the completed baseline questionnaires (i.e. smoking reduction or quitting, regular physical activity, healthy diet and reduce alcohol consumption). The subjects will also ask to state a targeted goal in which they perceive as the easiest to achieve, such as eating more vegetables, eating less salted or fried food, engaging in more or higher intensity of exercise, reducing alcohol consumption, reducing number of cigarette consumption per day or quitting. Each subject will then receive an individual face-to-face brief MI (about 5 min) with generic health advice on selected health-related lifestyle practice. All subjects will then be informed that they will receive an individual brief MI intervention to assist behavioural changes or achieve goals as desired or chosen by them via WeChat or WhatsApp in the smart phones throughout the study period. In addition, the subjects will be given a self-help smoking cessation booklet with a public quitline number.

#### Follow-up booster intervention

The trained research assistant will deliver brief MI to each subject in the intervention group individually via WeChat or WhatsApp in the smart phones throughout the study period. The brief MI messages will be delivered more intensively as preferred by the subject (usually not less than once per 2–3 days and no more than 2 times per day) for the first 6 months. The frequency of delivering message through WeChat or WhatsApp will be interactive, depended on subjects’ actions and responses. It may take several sessions of chats within several days/weeks. However, the total time spent by the research assistant would not be more than that for traditional MI with several long sessions.

Start from 6 months, minimal messages by merely following the subjects’ progress and responding to their questions to maintain contact will be provided to subjects till one-year follow-up.

#### Content of the brief MI messages

The content of the brief MI messages will depend on the desirable health-related lifestyle practice and the targeted goal in which the subjects perceive as the easiest to achieve. The trained research assistant will give brief MI messages with an aim at moving the subjects towards the goal. The brief MI messages will be guided by the menu of strategies (Table 1). As the trained research assistant moves down the menu, greater readiness to change from the subjects will be required. During the process of delivering brief MI via WeChat or WhatsApp, the trained research assistant will follow the menu of strategies and start from the top. To begin the conversation, the trained research assistant will first explore the subjects’ current barriers and facilitators, and lifestyles by asking, ‘What are your current lifestyle practice in general?’, and ‘What are your current barriers and facilitators?’ The trained research assistant will then raise the topic of not engaging in desirable health-related lifestyle practice with open-ended question such as ‘How does your current lifestyle practice fit in? ‘How does your current lifestyle practice affect your health?’ After that, the trained research assistant will ask the subjects to describe a typical day with how the current lifestyle practice fit in. In this strategy, it is important to train the research assistant to be aware not to push the subjects nor to insert their own hypothesis into the conversation. The trained research assistant will then bring the pros and cons of the subjects’ current lifestyle practice by questions such as ‘What are some of the good things/less good things of your current lifestyle practice?’. When the subjects become curious about the current or the desirable health-related lifestyle practice, the trained research assistant will ask for permission before providing relevant information by questioning ‘I wonder if you would be interested in knowing … ’. The trained research assistant will elicit a discrepancy by asking an open-ended question such as ‘How would you like things to be in the future?’. To explore the concerns, the trained research assistant will raise questions, ‘What concerns do you have about engaging in desirable health-related lifestyle practice?’ and ‘What other concerns do you have now?’. When the concerns are clearly manifest, an open question like, ‘What does this leave you now?’ can help subjects to decide the future actions. The research assistant will be trained to summarize subjects’ statements constantly throughout the conversation.

**Table 1:** The menu of strategies in brief MI

Strategies	Aims	Examples of questions
1. Opening strategy: lifestyle, stresses and current lifestyle practice	To establish rapport and understand the context of the current lifestyle practice	‘What are your current lifestyle practice in general?’, ‘What are your current stressors?, ‘Where does your current lifestyle practice fit in?’
2. Opening strategy: health and current lifestyle practice	To build rapport and relate health with the current lifestyle practice	‘How does your current lifestyle practice fit in?’, ‘How does your current lifestyle practice affect your health?’, ‘How do you think your current lifestyle practice relate to your health?’
3. A typical day	To further establish rapport, assist the subject to talk the current lifestyle practice in detail and to assess the readiness to change	‘Can you tell me about your day today from beginning to end?’, ‘Can you describe to you a typical day of yours, like today, what happened, how did you feel and where did your current lifestyle fit in? May we start from the beginning?’
4. The good things and the less good things	To continue establishing rapport, understanding the context of the current lifestyle practice, and minimize resistance in subjects as conversations will be started with positive points	‘What are some of the good things/less good things of your current lifestyle practice?‘, ‘What do you like/dislike about your current lifestyle practice?’
5. Providing information	To provide relevant information on the healthy lifestyle practices in a sensitive manner	‘I wonder if you would be interested in knowing …’, ‘I wonder if you would like to know more about …’, ‘I wonder what would you do after knowing …’, ‘How does these relate to your current lifestyle practice?’
6. The future and the present	To create discrepancies to motivate the subjects adopting the healthy lifestyle practices	‘How would you like things to be in the future?’, ‘What is stopping you from doing these things?’.
7. Exploring Concerns	To help subjects identify and explore concerns about adopting the healthy lifestyle practices	‘What concerns do you have about engaging in desirable health-related lifestyle practice?’, ‘What other concerns do you have now?’, ‘What else, what other concerns do you have?’
8. Helping with decision-making	To assist subjects in decision-making to adopt the healthy lifestyle practices	‘What does this leave toy now?’, ‘What are you planning to do now?’, ‘What are you going to do now?’

Also, the technique of expressing empathy will be incorporated in the intervention so as to develop a genuine and closer relationship of caring with the subjects. Expressing empathy is a specifiable and learnable skill for understanding another’s meaning through the use of reflective listening [16]. To incorporate such skill into the intervention, the trained research assistant will be reminded to show respect in the messages, especially not to impose direction and judgment regarding the subjects’ decision. Also, throughout the conversations, the attitude of the research assistant should be acceptance, but not necessarily approval or agreement. Besides, the research assistant will be reminded that ambivalence is normal and they should avoid forcing the subjects to change [16].

#### Assessment for readiness to quit at 3-month follow-up

Although subjects do not have an intention to quit smoking at baseline (one of the inclusion criteria), the readiness of quitting smoking will be assessed at 3-month follow-up. For those who are willing to take further actions to promote their health, i.e. with an intention to quit smoking, health advice on smoking will be given with more emphasis on the health benefits of quitting. Upon request by subjects, we shall provide them with more comprehensive information on quitting. Specifically, the intervention should address the needs of smoking patients by teaching them with the skills to overcome withdrawal symptoms or cigarette cravings. In addition, subjects will be allowed to select their own schedules of quitting, such as to quit immediately or progressively. Our previous randomised controlled trial on the effectiveness of a self-determination intervention for smoking cessation (immediate or progressive) among people attending emergency departments showed that by giving autonomy for smokers to select their own schedules of quitting would enhance their self-efficacy and competence in quitting smoking. For those who request nicotine replacement therapy (NRT) to assist them quitting, we shall refer them to a smoking cessation hotline in which free sample of NRT will be offered. The whole intervention will be given to the subjects through WeChat/WhatsApp during the process of the study. However, we understand that older people always appreciate regular phone conversations intermingled with message. Hence, there will be a follow-up telephone call with our subjects each month for the first 6 months so as to further build a closer relationship with them.

#### Intervention fidelity

A registered nurse with more than 5 year of experience in delivering smoking cessation interventions will be employed as the research assistant for this study. This registered nurse has already trained to use brief MI to provide face-to-face smoking cessation advices. However, to ensure that she will fully comply with our protocol, a half day training workshop by the research committee (principal and co-investigators) will be offered to her before this study. The workshop will include the principles and practices on brief MI and healthy lifestyles. The skills of brief MI will be co-learnt between the registered nurse and the principal and co-investigators through demonstrations and return demonstrations. Significantly, case study examples will be thoroughly discussed via role playing to permit the registered nurse to understand and practise the required skills. To ensure the quality and competence of the registered nurse in applying the learnt skills before implementation, she will be needed to complete an assessment with a case study examination after the workshop. The assessment will be performed by one of the research committee members who has expertise in using brief MI.

### Control group

Similar to the intervention group, the trained research assistant will first ask the subjects about the priority of engaging in any desirable health-related lifestyle practice and to state a targeted goal in which they perceive as the easiest to achieve after completing the baseline questionnaires. In addition, the subjects will be given a self-help smoking cessation booklet with a public quitline number. However, subjects in the control group will not receive brief MI and follow-up booster intervention.

### Data collection

#### Baseline

Before randomisation, the subjects will be invited to complete a structured questionnaire, administered by a trained research assistant face-to-face. This structured questionnaire will be developed through adopting or modifying international and/or locally validated instruments. The questionnaire will gather information including smoking and quitting history, stage of readiness to quit, utilization of existing smoking cessation services, and demographic information such as age, gender, and marital status. The clinical information will be obtained by the trained research assistant from the subjects’ medical records.

#### Follow-up

All subjects will receive follow-up telephone call at 1, 3, 6 and 12 months from our trained research assistant. In each telephone follow-up, the subjects will be asked to complete the structured questionnaire again. Subjects who are abstinent from cigarette use ≥7 days at 12-month follow-up will be invited for a biochemical validation. The biochemically validated 7-day point prevalence of abstinence will be confirmed by saliva cotinine level < 115 ng/ml in parallel test and a carbon monoxide level in expired air < 9 ppm (p.p.m.) [18]. These biochemical validation methods have been used in previous studies on smoking cessation [19, 20]. For subjects on nicotine replacement therapy, biochemical validation will be conducted 7 days after the completion of therapy.

### Outcomes

The primary outcome is biochemically validated smoking abstinence at 12 months.

Secondary outcomes are: (i) self-reported 7-day point prevalence of smoking abstinence at 6 and 12 months, (ii) self-reported reduction of ≥50% in cigarette consumption at 6 and 12 months, and (iii) any behaviour change as indicated by the subjects.

### Data analysis

The SPSS for Windows (version 25) will be used to conduct data analysis. We will first compare the baseline characteristics of the 2 groups using chi-square test for categorical variables and student t-test or Wilcoxon rank-sum test for continuous variables. The primary analysis will be the effect size of the intervention at 12 months. It will be estimated by the odd ratios (ORs). Crude ORs for quitting at 12 months will be estimated using logistic regression model and compared with ORs adjusted for potential or confirmed confounding baseline variables. The difference in biochemical validation quit rates at 12-month follow-up between intervention and control groups will be determined using Pearson’s chi-square test or with the use of Fisher’s exact test. Independent samples t-test will be used to determine whether there are statistically significant differences in length of abstinence for quitter between the intervention and control groups at 6 and 12-month follow-ups. The approach of intention-to-treat will be adopted. Those who are lost to follow-up or refuse to participate in the validation tests, will be treated as smokers with no reduction in cigarette consumption compared with (a) baseline, as the main analysis (by intention to treat), (b) the most recent level and (c) complete case (per protocol) analysis by excluding subjects with missing data as sensitivity analyses. Also, the number of communications with the subjects using WhatsApp/WeChat will be documented and analysed. In particular, all posts in the WhatsApp/WeChat will be archived. Each post will then be coded by 2 researchers independently, and will be classified by their content. Mann-Whitney U test will be applied to compare the median number of posts between the experimental and control group.

### Ethical consideration

This study was approved by the institutional Review Board of the Hospital Authority of New Territories West Cluster (reference: NTWC/REC/19001), and was registered on the ClinicalTrials.gov (reference: NCT03983330). During subject recruitment, all eligible smokers who are willing to participate will be briefed on the purpose, design, procedures, potential benefits and risks of the study. Written consent will then be obtained.

### Patient and public involvement

Patients and public will not be included in the development, design, implementation or dissemination of the research. The results will be disseminated to the subjects through patient forums.

## Discussion

To the best of our knowledge, this is the first study to determine the potential efficacy for the use of a personalized general health promotion approach in promoting smoking cessation for smokers with chronic diseases. In fact, using brief advice or counselling was the most common smoking cessation intervention for smokers with chronic diseases [5, 7, 8]. Although such brief interventions are more feasible in busy clinical settings, they may not strong enough to make a significant impact on smokers with chronic diseases [8], especially most of them do not have any intention to quit and with a long smoking history [5–7]. Hence, there is an imperative need to develop an innovative method to assist smokers with chronic disease to achieve abstinence.

This study has several strengths. Firstly, previous studies indicated that smoking was likely to coexist with other risk behaviours [9–11]. Smokers usually engaged in other unhealthy habits, i.e. physical inactivity, alcohol drinking and unhealthy diet [21]. If our proposed intervention is effective, we will able to assist smokers with chronic disease to quit smoking and change their health behaviour simultaneously. This can have a significant effect on health care because we will help improve the physical well-being of smoking patients and eventually save more lives. Secondly, although a majority of smokers with chronic disease do not intend to quit smoking and refuse to join any smoking cessation intervention [5–7], they are willing to improve their health [12, 13]. If this intervention approach is feasible, we will be able to build and maintain rapport with these smokers through providing advices on how to change their desirable health behaviours. Once they are prepared for quitting, we can then deliver smoking cessation advices. Thirdly, our proposed intervention will be delivered using information communication technology. In comparison to the face-to-face method, which is known to be time-consuming and labour-intensive, our results will generate evidence to support the use of information communication technology to deliver brief motivational interviewing to promote smoking cessation. The sustainability of this inexpensive method will be ensured as it can reach many smokers at a low cost. Besides, based on the findings of this randomised controlled trial, a large randomised controlled trial will be conducted to evaluate the effectiveness and costs of a personalized general health promotion approach in promoting smoking cessation for smokers with chronic diseases in the future.

## Data Availability

Not applicable.

## Abbreviations

SOPC: Special Out-Patient Clinics
MI: Motivational interviewing
SNOSE: Serially Labelled, Opaque and Sealed Envelope
NRT: Nicotine Replacement Therapy
ORs: Odd Ratios
